# Dermal Substitutes Use in Reconstructive Surgery for Skin Tumors: A Single-Center Experience

**DOI:** 10.1155/2017/9805980

**Published:** 2017-07-02

**Authors:** Mariane Campagnari, Andrea S. Jafelicci, Helio A. Carneiro, Eduard R. Brechtbühl, Eduardo Bertolli, João P. Duprat Neto

**Affiliations:** ^1^Skin Cancer Department, AC Camargo Cancer Center, São Paulo, SP, Brazil; ^2^Surgical Oncology, AC Camargo Cancer Center, São Paulo, SP, Brazil

## Abstract

Reconstructive surgery following skin tumor resection can be challenging. Treatment options after removing the tumor are skin grafting, local pedicled and axial flaps, or microsurgery for complex and extensive wounds correction. Recently, the use of dermal substitutes has been extended to reconstructive surgery in cutaneous oncology.* Objectives*. To report both a single-center experience using dermal substitutes in reconstructive surgery for skin malignancies and reconstructive surgery's outcomes.* Methods and Results*. Among thirteen patients, seven (53.8%) were male with mean age of 62.6 years. Regarding diagnosis, there were five cases (38.5%) of basal cell carcinoma (BCC), two (15.4%) of melanoma in situ, two (15.4%) of dermatofibrosarcoma protuberans, one (7.7%) of squamous cell carcinoma (SCC), one (7.7%) of angiosarcoma, and one (7.7%) of eccrine carcinoma (EC). The most common site of injury was scalp (53.8%) and lower limbs (23.1%). Seven (53.8%) patients used NPWT and six (46.2%) patients underwent Brown's dressing. The most frequent complication of the first stage was wound contamination (38.5%). Average time to second-stage skin grafting was 43.9 days. Three (23%) patients developed tumor recurrence and one died.* Conclusions*. Use of dermal substitutes in oncology can be an option for reconstruction after extended resections, providing good aesthetical and functional results.

## 1. Background

Reconstructive surgery following skin tumor resection can be particularly challenging due to the nature of disease process, including the extent of resection, need for preserving original surgical margin orientation, possibility of tumor recurrence, and requirement for adjuvant radiation therapy. Moreover, following tumor resection, reconstruction must be tailored to clinical prognosis, treatment plan, patient's age, and desired cosmesis [1].

Treatment options after tumor removal are skin grafting, local pedicled and axial flaps, or microsurgery for complex and extensive wounds correction. Each technique has not only its advantages but also its limitations.

Dermal substitutes such as Integra® and Matriderm® were initially used for treating burns and were later extended for reconstructive surgeries and treatment of chronic wounds [1–5].

Integra (LifeSciences Corporation, NJ, USA) is a bilaminate synthetic construct consisting of an outer silicone layer and an inner collagen-glycosaminoglycan matrix. The first layer is a matrix of bovine collagen and chondroitin-6-sulfate, cross-linked with glutaraldehyde; after grafting, recipient fibroblasts infiltrate the matrix network and synthetize a neodermis, which is histologically very close to normal human dermis. The second layer is a silicone membrane, which acts as a temporary epidermis [6, 7].

Matriderm (Dr. Suwelack Skin & Health Care AG, Billerbeck, Germany) is a single layer composed of bovine collagen and elastin. The collagen promotes rapid cell migration, proliferation, and revascularization and the elastin encourages early neoangiogenesis and elastin synthesis [8, 9].

More recently, Matriderm and Integra use in oncology and reconstructive surgery has also been reported [10–12]. These reports show no difference in oncological outcomes and are technically feasible. In addition, good aesthetical results have been achieved.

## 2. Objectives

We aim to report a single-center experience using dermal substitutes in reconstructive surgery for skin malignancies and the outcomes of those surgeries.

## 3. Patients and Methods

We present the report of thirteen patients treated in the Skin Cancer Department of AC Camargo Cancer Center, São Paulo/SP, Brazil, who underwent reconstructive surgery with artificial dermis between December 2012 and November 2016. All patients were informed before surgeries, and an informed written consent was provided.

Clinical data was analyzed regarding age, tumor type and location, tumor size in pathology report, comorbidities, postoperative complications, and recurrence. Reconstruction was carried out in a two-stage process and the time interval between them was also analyzed. One patient underwent only first-stage surgery, due to complete epithelialization. Another patient is still waiting grafting after multiple debridement surgeries of devitalized and necrotic tissue.

**Figure 1:**
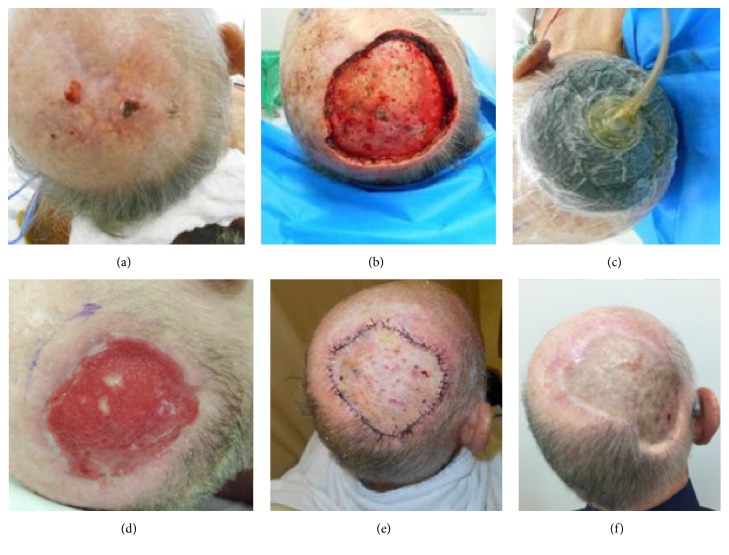
(a) Basal cell carcinoma in scalp, clinical aspect before surgery; (b) surgical bed with periosteal removal; (c) negative pressure wound therapy was applied after Matriderm to enhance granulation; (d) granulation tissue; (e) skin graft, early postoperative aspect; (f) late postoperative aspect.

First stage involved removal of tumor and application of artificial dermis. Frozen section exam was performed to ensure clear margins, except both in DFSP cases, in which circumferential and deep margin assessment is the standard of care, and in melanoma cases. First stage was followed by Brown's dressing or Negative Pressure Wound Therapy (NPWT) (Figure 1). Our intention was to use NPWT in every patient. However, some of them could not use it due to local pain or in some specific locations such as nail unity. Besides that, some patients had no coverage of their health insurances to this product.

Brown's dressing was removed 5 to 7 days after surgery and new dressings were performed three times a week with Mepilex Ag™ (Mölnlycke Health Care, Göteborg, Sweden) or PolyMem Silver™ (Ferris Mfg. Corp., TX, USA) to help control local contamination. Patients with NPWT were evaluated weekly, following the same procedures as mentioned below. Wound was inspected to assess for contamination or local complications until a well-vascularized neodermis was achieved. Second stage was only performed when the wound showed adequate granulation tissue and subsequently covered with a split-thickness skin graft.

Clinical follow-up of patients was done in outpatient clinics periodically to assess treatment plan, tumor recurrence, and durability of the reconstruction.

## 4. Results

Clinical data is summarized in Table 1. Mean age was 62.6 years (range 39 to 86 years). Cohort comprised seven (53.8%) male and six (46.2%) female patients. Only two patients had no comorbidities. The most common comorbidities were high blood pressure (46.2%), heart disease (30.8%), former smoking (30.8%), hypothyroidism (23.1%), diabetes mellitus (15.4%), and smoking (7.7%).

Regarding diagnosis, five (38.5%) patients presented with basal cell carcinoma (BCC), all of them with sclerodermiform subtype, two (15.4%) with melanoma in situ, two (15.4%) with dermatofibrosarcoma protuberans, one (7.7%) with eccrine carcinoma, one (7.7%) with squamous cell carcinoma, and one (7.7%) with angiosarcoma. One patient presented with radionecrosis after treatment for malignant fibrohistiocytoma and another used dermal substitutes in the donor area of a frontal skin flap. The most common site of injury was the scalp (53.8%) followed by lower limbs (23.1%) and fingers (15.4%).

Only one patient used Integra. All the others used Matriderm 2 mm. Seven (46.2%) patients used NPWT after first surgery. However, one patient could not tolerate NPWT and had it removed after first week due to local pain. Six (46.2%) patients underwent Brown's dressing.

Average time to second-stage skin grafting was 43.9 days (range 28 to 64 days). The most frequent complication of first stage was wound contamination (38.5%), which was treated with Mepilex Ag or PolyMem Silver and oral antibiotics. Regarding the second stage, partial loss of the graft occurred twice, treated with the same dressing.

Three (23%) patients developed tumor recurrence. And among patients who underwent skin grafting, two (18.2%) experienced partial loss of the graft. One patient did not require a second-stage reconstructive surgery and the other one is still unable to undergo grafting. One patient died due to disease progression. Three patients were lost to follow-up.

Radionecrosis patient's granulation process in surgical bed was slow, which made grafting unfeasible after first surgery. This patient required other surgeries for debridement of devitalized and necrotic tissue. Currently, granulation tissue presents good aspect and skin grafting is being scheduled.

## 5. Discussion

Reconstructive surgery plays a very important role in cutaneous oncology. Several skin malignancies may lead to complex defects and, therefore, complex reconstructions.

Skin graft may be the simplest option. It is associated with minimal donor-site morbidity and is cost-effective but can be prone to contraction and suboptimal aesthetic appearance [13–15]. In particular skin graft is unreliable on the previously irradiated wound bed and should be avoided whenever there are bones, tendons, and nerves exposed [6].

Locoregional flaps particularly in the scalp and lower limbs can cover exposed bone or tendons but are limited by the size of the defect. Also, the donor site may require grafting. Previous irradiation of tissues can lead to poor take of the flap or delayed healing. Local pedicled and axial flaps are more tolerant to therapeutic radiation. Moreover, the choice of free flap requires the presence of a long vascular pedicle and an adequate surface area of the flap [1].

Free tissue microvascular reconstruction of oncological defects, particularly in larger defects, remains the gold standard for covering large tissue defects, with successful transplant rates ranging from 95 to 99 percent. However, the application of either free or pedicled vascularized tissue transfer is associated with substantial donor-site morbidity, prolonged operative time, and hospital stay [1]. Also, it requires appropriate equipment and enabled professionals.

Artificial dermis is an alternative for the treatment of complex wounds, as it allows their closure with less morbidity and surgical time. Artificial dermis offers lower wound contraction, improved elasticity, and skin thickness in relation to grafts. It is also a simple procedure when compared to microsurgical flap and can be performed in previously irradiated areas, allowing wound cover in complex resection areas, better local control of the disease, and early detection of recurrence. The need for experienced surgeons, weekly returns to dressing change, high product cost, and inability to use artificial dermis on wounds with exposed dura mater [5, 7, 10] can be pointed out as disadvantages.

**Figure 2:**
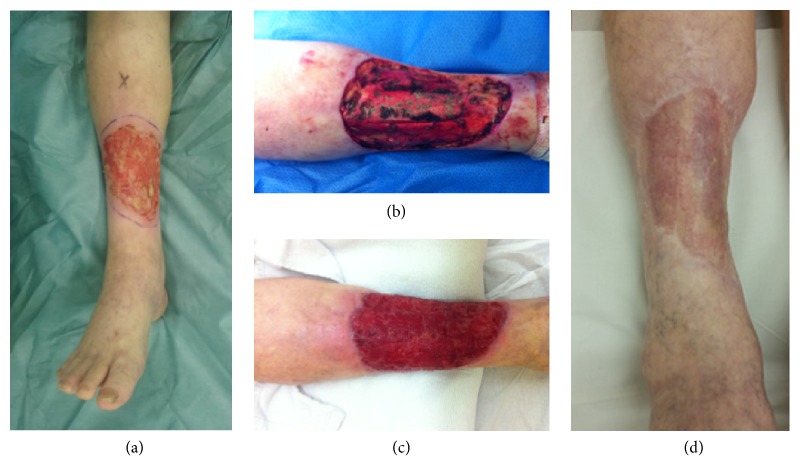
(a) Basal cell carcinoma in right leg, clinical aspect before surgery; (b) periosteal removal was necessary to ensure deep margins, which would not allow skin graft; (c) granulation tissue 28 days after Matriderm was placed (no negative pressure wound therapy was used in this case); (d) late postoperative aspect.

In summary, the choice of reconstruction is influenced either by patient factors such as age, coexisting medical conditions, length of procedure, and performance for general anaesthesia, as by surgeon's experience and surgical equipment, and/or material availability. Our experience with dermal substitutes is presented since we believe it is a well-tolerated option for the patient, with no prejudice to oncologic principles (Figure 2). We used it in more aggressive tumors such as angiosarcoma and eccrine carcinoma, and both can be easily followed up either by clinical examination or by radiologic exams.

There were three recurrences in our cohort. One of them, the eccrine carcinoma (patient 1), died due to complications of chemotherapy after disease progression. The second case experienced recurrence in scalp and underwent salvage surgery. The third one relapsed in transit disease and parothid and is currently on Vismodegib. They were both diagnosed with sclerodermiform BCC with perineural invasion.

Regarding clinical associated conditions, smoking and diabetes mellitus are known to have higher rate of surgical complications. In this study, the only smoking patient had no wound contamination, progressing with partial loss of the graft after second surgery. Longer dressing care was needed to this patient without prejudice to the aesthetic and functional results. Patients with diabetes mellitus or former smoking showed no surgical complication. No patient had prohibitive risk for anesthetic procedure.

In our data, there were five cases of wound contamination at the first stage. One patient had purulent discharge and necrotic wound, treated with debridement, washing, and dressing with Mepilex Ag or PolyMem Silver and oral antibiotics. Grafting was undergone successfully, but after five months, the patient developed tumor recurrence (eccrine carcinoma) and once again underwent resection of the tumor and reconstruction with regional flap.

The other four patients had only serohematic/serous secretion and underwent only dressing changes before second-stage surgery. Eleven patients underwent grafting in the second stage and only 2 (18.2%) had partial graft loss, but without aesthetic or functional impairment.

In our cohort, the most frequent skin malignancies were sclerodermiform BCC and DFSP, which are frequently associated with wide excisions due to the risk of local recurrence [16]. We also had the scalp as the most prevalent body region, which may also lead to a more challenging reconstruction.

Scalp defects can also be covered using delayed local flaps and tissue expansion. However, in the oncological patient who requires resection, tissue expansion should be used carefully as a reconstructive option since the tumor resection is not delayed because of use of the expansion [6, 17].

**Figure 3:**
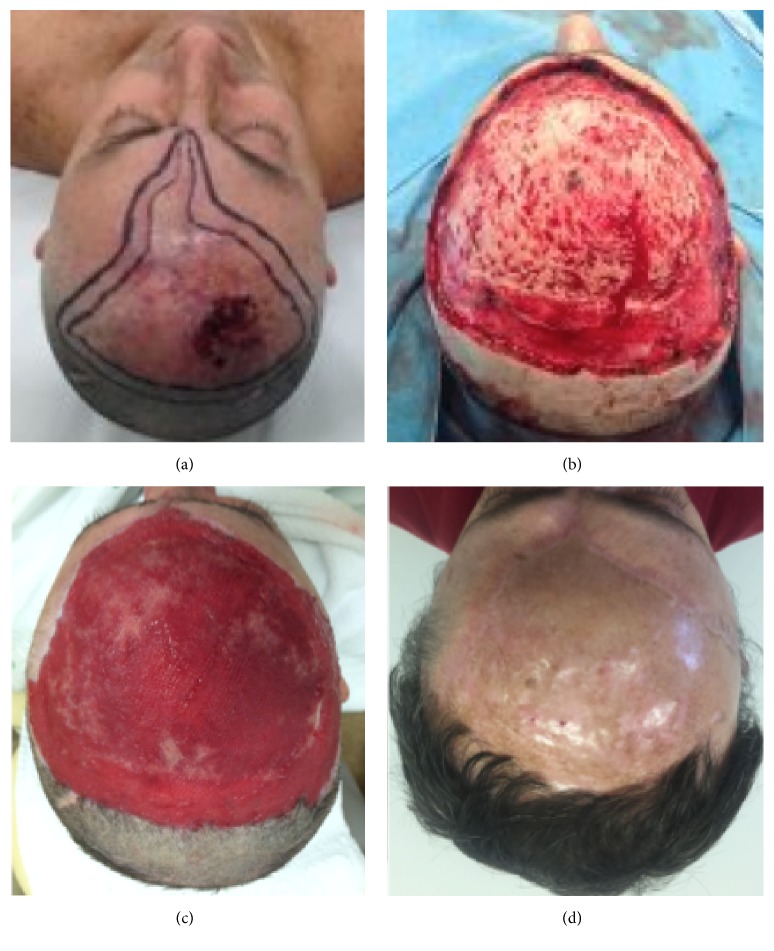
(a) Basal cell carcinoma, clinical aspect before surgery; (b) once again, periosteal removal was necessary; (c) granulation tissue (negative pressure wound therapy was used); (d) late postoperative aspect.

As an example, we can mention patients 4 (Figure 3) and 7 (Figure 1) who underwent resection of BCC on the scalp. In both patients, wide excision of the tumor was performed, which was more extensive in patient 4, who needed to have the bone drilled due to involvement of the periosteum.

As reported in other studies, it is known that bone drilling is an important tool used in periosteal involvement of cases to ensure safety margin of tumor resection and even in cases of extensive lesions providing vascularized area to the fixing template. In our data, five (71.4%) patients underwent drilling of scalp injury, due to periosteum involvement by the tumor, to hasten granulation process. The two remaining (28.6%) patients required only periosteum excision.

Two patients had in situ acral melanoma. Digital sparing surgery in this situation can be an option. However, in this scenario, reconstruction after wide local excision can be associated with functional loss or chronic pain. In such cases, dermal matrix plays an important role as it provides satisfactory aesthetic and functional outcomes [12]. In both cases, amputation could be avoided and the patients report good quality of life after surgery.

Fixing the artificial dermis in the surgical bed can be done either with Brown's dressing or with NPWT. Brown's dressing promotes intimate contact of template with bloody area, providing adequate fixation thereof to wound with tissue neovascularization and granulation tissue. In our routine, we evaluate granulation tissue 3 times a week and Mepilex Ag or Polymem Silver dressings are changed whenever necessary. NPWT can help remove deleterious substances from the wound, relieve edema, and stimulate cell proliferation, thereby promoting granulation and inhibiting chronic inflammation. Furthermore, NPWT also seems to assist in neovascularization of skin grafts and tissue-engineered skin substitutes and can improve the success rate of skin grafting by strengthening bonding between the skin graft and the recipient area. Additionally, NPWT must be evaluated weekly [18].

NPWT was used on 5 patients with lesions of scalp due to extension of the lesion in order to enhance granulation and subsequent grafting. The average time for grafting in these patients was 49.6 days. Also, we used NPWT in 2 patients with lower limbs injury. One patient did not tolerate the pain and the other patient could undergo grafting in 36 days. One patient who underwent resection of a nail melanoma and used Brown's dressing did not require grafting, due to complete epithelialization after 90 days. Average time for grafting in five patients with Brown's dressing was 39.8 days.

There are evidences in literature that the use of vacuum dressing decreases the time required for formation of granulation tissue and subsequent grafting [19–21]. However, in our cohort, patients who used NPWT took on average 47.3 days to undergo the second surgery compared to patients who used Brown's dressing with an average of 39.8 days.

We believe that this can be explained by the greater extension of the lesions and the exposed bone. Although our sample is insufficient for statistical validation, we also believe that, even in the absence or inability of NPWT use, Brown's dressing is an acceptable option for the use of dermal substitutes in the reconstructive setting, especially in smaller defects.

Two patients did not achieve second surgery. One patient had complete wound epithelialization with artificial dermis. The other one presented with a complex wound secondary to radionecrosis in her leg after radiotherapy for a malignant fibrohistiocytoma treatment.

**Figure 4:**
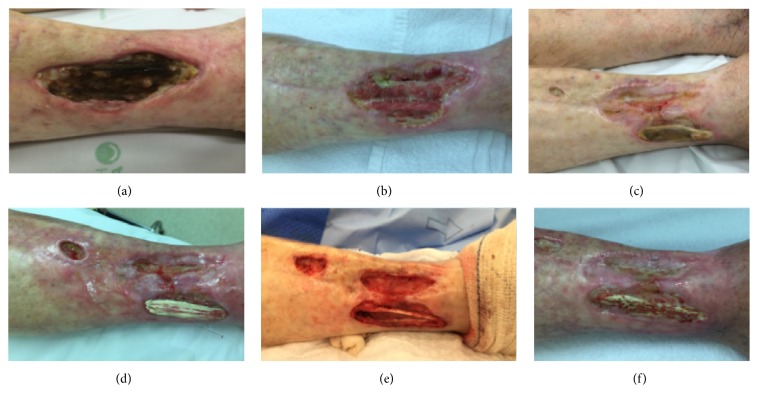
(a) Radionecrosis in right leg, clinical aspect before first surgery; ((b) and (c)) there was no granulation in the area of exposed and necrotic tendon, which led to ((d) and (e)) debridement and removal of necrotic tendon. This was the third time that Matriderm was placed; (f) clinical aspect, waiting for skin graft.

She underwent three surgical procedures for debridement of devitalized and necrotic tissue, requiring resection of the exposed necrotic tendon in the last procedure. Subsequent placement of dermal matrix was performed in all surgical approaches to enhance the formation of feasible tissue to carry out grafting. After her last surgery, the patient has been developing good but still slow formation of granulation tissue (Figure 4). Skin graft is to be scheduled.

There is no report in literature of dermal substitutes use in radionecrosis. However, we believe that, in these complex wounds, dermal matrix could be an ally in its treatment. It could enhance granulation tissue formation for subsequent grafting with an adequate aesthetic and functional result.

We present similar data to what is reported in literature. The use of dermal substitutes in oncology can be an option for reconstruction after extended resections, providing good aesthetical and functional results. Therefore, it can be performed within a shorter period of time, which is useful in patients with clinical comorbidities, such as the ones we had in our patients. Further studies are necessary to validate this procedure.

## Figures and Tables

**Table 1:** Clinical features and outcomes summary of the patients who underwent reconstructive surgery with dermal substitutes in AC Camargo Cancer Center, from 2012 to 2016 (M = male, F = female, EC = eccrine carcinoma, DFSP = dermatofibrosarcoma protuberans, BCC = basal cell carcinoma, SCC = squamous cell carcinoma, MMis = malignant melanoma in situ, STSG = split-thickness skin graft, N/A = not available, and *∗* size according to pathology reports, without considering further excisions after frozen section when needed).

Patient	Age	Sex	Abnormality	Tumor location	Size (cm)	Comorbidity	Dressing	Complications after 1st surgery	Application of STSG (days)	Complications after 2nd surgery	Follow-up
1	64	M	EC	Scalp	20,5 × 20	Hypothyroidism Heart disease Rheumatoid arthritis	Brown	Wound secretion/debridement	28	Partial graft loss	Death

2	56	M	DFSP	Scalp	14 × 13	High blood pressure Obesity Former smoking	Vacuum	Wound secretion	50	—	No recurrence

3	84	F	BCC sclerodermiform	Lower limbs	13,8 × 8,6	Heart disease Former smoking Alzheimer's disease	Brown	—	29	—	No recurrence

4	51	M	BCC sclerodermiform	Scalp	N/A	—	Vacuum	Wound secretion	50	—	Parotid metastasis In transit disease

5	56	M	BCC sclerodermiform	Nose	5 × 4,5	High blood pressure Smoking	Brown	Wound secretion	57	Partial graft loss	Lost

6	65	M	MMis	Digital	3,5 × 2,5	Former smoking Chronic obstructive pulmonary diseases	Brown	—	Unnecessary	—	Lost

7	75	M	BCC sclerodermiform	Scalp	8 × 5	High blood pressure Former smoking	Vacuum	—	41	—	Recurrence at 20 months

8	50	F	DFSP	Scalp	7 × 5,5	—	Brown	Wound secretion	35	—	Lost

9	77	F	Radionecrosis	Lower limbs	N/A	High blood pressure Heart disease Hypothyroidism	Vacuum	Slow granulation	Not grafted	—	No recurrence

10	41	F	MMis	Digital	3 × 3	Hypothyroidism	Brown	—	50	—	No recurrence

11	39	F	SCC	Lower limbs	13 × 6	High blood pressure, diabetes mellitus, multiple sclerosis	Vacuum	—	36	—	No recurrence

12	70	M	Angiosarcoma	Scalp	5,2 × 4,5	Parkinson's disease	Vacuum	—	43	—	No recurrence

13	86	F	BCC sclerodermiform	Scalp	12 × 12	High blood pressure Heart disease Diabetes mellitus Renal chronic failure	Vacuum	—	64	—	No recurrence

M = male, F = female, EC = eccrine carcinoma, DFSP = dermatofibrosarcoma protuberans, BCC = basal cell carcinoma, SCC = squamous cell carcinoma, MMis = malignant melanoma in situ, STSG = split-thickness skin graft, and N/A = not available.
